# Book Reviews

**Published:** 1876-02

**Authors:** 


### Book Reviews.

[NOTE. — All works reviewed in the pages of the CHICAGO MEDICAL JOURNAL AND EXAMINER may be found in the extensive stock of W. B. KEEN, COOKE & Co., whose catalogue of Medical Books will be sent to any address upon request.]

A TREATISE ON THERAPEUTICS, Comprising Materia Medica and Toxicology, with Especial Reference to the Application of the Physiological Action of Drugs to Clinical Medicine. By *H. C. Wood, Jr., M. D.*, Professor of Botany and Clinical Professor of Diseases of the Nervous System in the Med. Dept. of the University of Pennsylvania, etc., etc. Second Edition, Revised and Enlarged. Philadelphia: J. B. Lippincott & Co. 1876.

The appearance of the second edition of this work within a twelvemonth of the issue of the first edition, is a quiet testimony to the superiority of the volume, which needs no commentary or qualification. This book contains about 150 pages more than its predecessor. The subjects additionally treated of, are Coffee and Tea, Tobacco, Arnica, Eucalyptus, Picric Acid, Lithium, Oxalate of Conium, Gelseminum, Jaborandi, Salicylic Acid, Cold and Heat, and Electricity.

The author in his preface says that, "The old and tried method in therapeutics is that of empiricism, or, if the term sound harsh, of clinical experience." * * "The plan of the present work has been to make the physiological action of remedies the principal point of discussion. A thoroughly scientific treatise would, in each article, simply show what the drug does when put into a healthy man, and afterwards point out to what diseases or morbid processes such action is able to afford relief. Unfortunately our knowledge is not complete enough for this, and the clinical method has to be used to supplement the scientific plan." As a result of this plan, we are treated to the physiological actions of all the drugs that have been experimented with up to the present time. These drug experimentations have been carried on extensively by the author and by European physiologists. This treatise is made up largely of the results of these observations, collected from English, French, Italian, German and Russian sources. *The* distinctive feature of each article of the materia medica treated of by Prof. Wood, is its physiological action. Alcohol, *e. g.*, has eight pages devoted to its physiological action and three pages to its therapeutical uses. The article on Quinia has eleven pages on the physiological action, and only six pages on its therapeutical uses. These minute and extended accounts of this action very naturally lead one to suppose that there must be some sort of connection between the physiological action and the therapeutical use. This connection is not always apparent, and in very many places where the former would suggest the latter, not one word appears to confirm some of the very numerous *new* therapeutical ideas which are now taught in our colleges and practiced extensively. To illustrate this point, we refer to Corrosive Sublimate. Prof. Wood's article is limited to about one page, detailing the physiological action of this drug, and he has not one single suggestion to offer on the therapeutics of it. Producing such disastrous effects on the alimentary canal as this agent does, one would naturally expect some good might be extracted from it; and *experience* shows its incomparable advantages in dysentery, but our author does not allude to it. This book contains very much that is *more* than good, very much that is *most excellent*, yet we exceedingly regret to see our author confirming the reputation that Philadelphia physicians bear, of ultra conservatism in the uses of drugs. The only blemish that his really grand work bears, is the conspicuous absence of the thousand-and-one minor therapeutical points which characterize such modern works as Ringer's, Gubler's and Arinand de Fleury's.

The article on Electricity—most excellent in itself—is supplemented, in the appendix, with several cuts, illustrating the various points of the human body upon which to place the electrode to produce localized electrization of the muscles. We are exceedingly astonished to see the author utterly oblivious to the authorship of these illustrations: they are evidently copied, with exactitude, from "Ziemssen's Electricität in der Medicin, vierte, Umgearbeitete Auflage, 1872."

So complete a work as Prof. Wood's professes to be, only leaves one exceedingly desirous of the pleasure of perusing extended articles from his pen on such subjects as the elimination of drugs from the system, the differences of action between single and reiterated doses of drugs, the specialization of action upon the motor and secretory nerve centres of "Local Remedies," as our author calls them, and many other subjects of a kindred nature, suggested by his elaborate accounts of the physiological action of medicines.

A Practical Treatise on Diseases of the Eye. By *Robert Brudenell Carter, F.R.C.S.* London: Macmillan & Co. 1875.

This is a practical book indeed; and one which can be highly recommended to both the student and the practitioner. The author has very wisely omitted all theoretical speculations which abound in many continental works on the same subject; he has preferred to travel the safe road of well-settled facts and experience. With a view to practical instruction, he has not thought it necessary "to dwell minutely upon maladies of rare occurrence," nor has he attempted "to achieve that kind of completeness which is procured by undigested compilation." Desirous of giving the reader *his* experience, he very naturally has but little reference to modes of practice of which he is unable to speak from experience.

The book is composed of fifteen chapters. The first five chapters give an ably written account of the "Anatomy and Physiology," the "Examination of the Eye," the "Ophthalmoscope and its Application," the "Principles of Ophthalmic Therapeutics," and the "Principles of Ophthalmic Surgery." The other nine chapters are devoted respectively to the diseases of the eyelids, conjunctiva, cornea and iris: cataract, glaucoma, disorders of the fundus oculi, injuries, squint and paralysis, and the uses and selection of spectacles.

For the first time, we think, the interesting fact has been published (page 70), that prior to Helmholtz, in 1848, Charles Babbage constructed a perfect ophthalmoscope which, unfortunately for the scientific fame of England, remained unknown. In regard to the latest improvements of the ophthalmoscope, Carter says (page 95), "one of the most convenient forms is that of Loring as modified by Dr. Noyes of New York." We have not heard of an ophthalmoscope of Noyes, and are inclined to believe that the author meant to write "of Knapp," whose ophthalmoscope is superior to all, but is not mentioned in the book before us.

Carter is a strong advocate for the use of iodide of potassium and mercury in affections of the cornea and iris; and his remarks in regard to the choice between the two are very interesting (page 130): "It may be said that the best rule in practice is to inquire whether the local changes in actual progress are such as to inflict irreparable injury unless speedily arrested. If they are, iodide of potassium should be given in the first instance. If the changes in progress are not of this pressing character, it may often be best to give mercury from the first." The correctness of this view may be questioned by many, but all will fully indorse the following rule (page 140): "While we must neither overlook nor neglect to treat the systemic conditions on which affections of the eye may more or less depend, we must not place reliance upon constitutional treatment alone, to the neglect of the various local applications, by which the local malady can be conducted to a safe and speedy termination. Of the two errors, the less serious would be to neglect the constitutional treatment."

A good ophthalmic surgeon, according to the author, should have a high development of muscular sense, consentaneous action of both hands, ambidexterity, steadiness, and a great deal of preliminary practice in the dissecting room. The instruments, he divides into two classes (page 151): "the essential, which are representative of inventive ingenuity, and the superfluous, which are representative of inventive awkwardness. The former class have been contrived by good mechanicians; the latter by bad surgeons."

Although most operations on the eye are not exceedingly painful, he advises the use of an anæsthetic, and he gives his preference to sulphuric ether (page 180).

His abortive treatment of *styes* is described (page 196) thus: "as soon as the first stage of the coming stye is perceived, the pimple attended with a stinging sensation which the patient can at one recognize, the eyelash passing through this pimple should be extracted by means of proper forceps, and a very fine point of mitigated nitrate of silver should be immediately placed upon the mouth of the open follicle and pressed there for a few seconds."

The passage on "Blepharitis" (page 197) is one of the best in the book. But we are sorry to see the author write so discouragingly on strictures of the nasal duct (page 220): "I have come to the conclusion that the best treatment, in a great number of instances, is that which is only palliative, and that the best surgery, generally speaking, is to leave the obstruction alone." We hope none will imitate this "nihilistic" surgery.

The diseases of the conjunctiva, most practical of all practical subjects in ophthalmology, are not discussed as elaborately as their importance would deserve. Carter's treatment is based on the principles generally adopted. Only we do not rely as confidently as he (page 227) on a solution of nitrate of silver of the strength of two grains, in the treatment of the blennorrhœa neonatorum. With reference to pterygium we do not believe that (page 257) "the patient seldom derives material or lasting benefit from any operation;" nor can we sanction this advice, if "it extends so far over the cornea as to obstruct vision, * * then the best course is to enlarge the pupil by iridectomy."

In the treatment of diseases of the cornea, blunders are so exceedingly common that every student of medicine will do well to bear in mind these golden rules (page 271): "Keratitis, whatever else it may require, invariably requires local sedatives and local rest; and when it is even threatened, the instillation of atropine, the enforcement of functional disuse and the application of a protective bandage, should be regarded as necessary measures. In the somewhat hybrid cases in which there is unmistakable conjunctivitis, with a tendency to extension of vessels upon the cornea, it is often necessary to combine the different methods of treatment which are required for the two conditions. When this is done, too much care cannot be taken to keep the adstringent away from the cornea and to limit its action to the retro-tarsal fold. These may be touched with a stick of diluted nitrate of silver or of lapis divinus; but the application must be carefully neutralized or removed before the lids are suffered to return to their natural position."

In iritis, whether syphilitic or not, the author gives mercury on the following principle (page 314): "I accept resistance to atropine as the one sufficient proof that mercury is needed." "Nor can I believe that the rapidly beneficial action of mercury affords, in itself, any conclusive evidence of syphilis." "I have seen iritis, which I had every reason to think syphilitic, yield readily to atropine alone, and have seen iritis, which I had every reason to think non-syphilitic, resist atropine, and yield only to mercury."

Since Von Graefe introduced his "modified linear extraction" the operation for cataract has been the favorite topic among ophthalmic surgeons, and Carter also gives the subject a very elaborate discussion. He advocates discission for the cataract of infants, discission, combined with suction, for all cataracts in persons under thirty years, and Graefe's operation for cataracts of older patients. Very interesting are the author's remarks in reference to the "latest improvements" (?) by Kuchler, Liebreich and others.

Concerning the nature and treatment of the glaucomatous diseases, Carter points, with particular stress, to the intimate relation between glaucoma and morbid changes in the trifacial nerve. His advice to use atropine after the operations for glaucoma (page 422) sounds rather singular in the face of the well-observed fact that the use of atropine has in some eyes lighted up the glaucomatous process.

In regard to the diagnostic value of the "choked disc" as an evidence of some intra-cranial lesion, Carter is quite right in saying (p. 426), "I myself can only say that it is frequently associated with such lesion." And to substantiate his reserved opinion he relates the case of a little boy who had choked discs of the most typical character, and several ophthalmologists who saw him had no doubt that he was the subject of some form of brain disease; he died of pleurisy and advanced kidney disease, "and no trace of mischief in his brain could be discovered by the most careful examination," and at the same time a woman whose eyes presented the typical changes associated with albuminuria died, with healthy kidneys, of a tumor in the cerebellum.

His experience with strychnia for atrophy of the optic nerve is not very encouraging (page 436); and he is not able to say in which cases it may act beneficially and in which it will not, unless it has been tried for a few weeks. Such is our experience also; but when we use strychnia we would never dare to allow any member of the patient's family to make the subcutaneous injections, as does Carter.

With most English surgeons, so he employs Critchett's subconjunctival operation for squint, and he pronounces this method superior to that of Von Graefe "in every respect" (p. 507). This is his personal opinion which we do not share ; but we fully agree with him in saying (p. 505) : "The only method of perfectly curing squint, even when it is of a moderate degree, is to divide the correction as nearly as may be possible, equally between the two eyes, which really, though not apparently, participate equally in the deviation."

The discussion of "the use and selection of spectacles" is scientific, plain, and exceedingly practical, especially his remarks on the treatment of presbyopia and myopia. "I never recommend the use of weak common spectacles which leave the (presbyopic) patient struggling, as it were, on the very brink of his infirmity and compel him always to exert his ocular muscles to the full measure of their powers," (p. 543). And as to myopic persons, "the limited range of their vision is alone an evil of no small magnitude. Young persons who are short-sighted, and who are suffered to grow up without spectacles, that is to say, with no distinct vision of anything which is more than six inches or twelve inches from their noses, lose an amount of unconscious education which no teaching can supply," (p. 558).

The text is fairly illustrated with diagrams, which, we are free to say, are far more useful than the four engravings which are of very inferior workmanship. With its slight blemishes, the book is yet a valuable contribution to the library, an instructive guide to the learner, and an entertaining companion for the learned.

INSECTIVOROUS PLANTS. By *Charles Darwin, A.M., F.R.S., etc.* With Illustrations. Pp. 462. 8vo. New York: D. Appleton & Co. 1875.

This is the only book in our language treating of plants that catch, destroy, digest and assimilate insects. Naught but commendatory words can be spoken of it. The author's name is sufficient guaranty of its intrinsic merit and eminently scientific character. The detailing of observed facts in the history of these plants, is the characteristic of the work. Those particularly interested in the study of botany or comparative physiology will find this volume of great value.

A Course of Lectures on Physiology; as delivered by *Professor Kuss*, at the Medical School at Strasbourg. Translated from the Second and Revised Edition by *Robert Armary*, *M.D.*, formerly Professor of Physiology at the Medical School of Maine, etc. One hundred and fifity wood cuts; 8vo.; pp., 531. Boston: James Campbell.

So many works on physiology have appeared within the past seven years, that it may be invidious to make special mention of any new one, like the above; but, in all candor, any one reading Prof. Küss' lectures must acknowledge that he has a very happy method of presenting the science that he attempts to teach, and that he has kept his book fully up with the latest developments of this subject. As proof of the foregoing remark, the reader is referred, especially, to that part of the subject relative to the functions of the nervous system, in all of the physiological phenomena of the human body. The book is devoid of the exceedingly awkward arrangement of the text of Prof. Hughes Bennett's volume on Physiology; it is not so cumbersome as Flint's late work; and it is superior to Kirke's late work on this subject, in that it presents more details on the subject of alimentation. It is a book that can be heartily recommended to the student and practitioner. Dr. Armary may be congratulated on his selection of such an excellent treatise for translation.

Outlines of Practical Histology; being the Notes of the Histological Section of the Class of Practical Physiology, held in the University of Edinburgh. By *William Rutherford*, *M.D.*, *F.R.S.E.*, Professor of the Institutes of Medicine in the University of Edinburgh, Examiner in Physiology in the University of London.

A prominent divine, not far from Chicago, lately received the degree of LL.D., from an Eastern University, in addition to the burden of titles which he carried aforetime. In his letter acknowledging the honor, he wrote: "I seem to myself like an ancient lamp—pretty much all handle." So diminutive is Dr. Rutherford's book, and so ponderous the title, that one is almost warranted in saying, that it is "pretty much all handle." But it ought to be a very useful book after all. It is simply intended as a hand-book of elementary histology, "introductory to an advanced study of the subject;" but the student, and generally the physician, who "works out" the problems which Dr. Rutherford so laconically puts before him, will know a vast sight more of histology and microscopy than he did at the beginning. We know of no better manual for the student who has but little time to spend in practical laboratory work, and desires to use that little to the best advantage.

I. N. D.

MINOR SURGERY AND BANDAGING. By *Christopher Heath, F.R.C.S.* Philadelphia: Lindsay & Blakiston.

This useful little manual, addressed to the wants of the student and young practitioner, has passed to the fifth edition. In the work referred to, Mr. Heath's primary object has been to offer to the house surgeon some serviceable hints on the management of the many accidents and emergencies daily coming under his care. The author has been particularly happy in condensing much useful matter into small compass, and we take pleasure in recommending the work to the house surgeons of hospitals, to students, and those who desire to acquire a knowledge of the little things in surgery—details, upon which, so often, success depends. J. E. O.

